# Performance of MALDI biotyper compared with Vitek^™^ 2 compact system for fast identification and discrimination of *Staphylococcus* species isolated from bovine mastitis

**DOI:** 10.1002/mbo3.389

**Published:** 2016-07-01

**Authors:** Ayman Elbehiry, Musaad Al‐Dubaib, Eman Marzouk, Salama Osman, Husam Edrees

**Affiliations:** ^1^ Department of BacteriologyMycology and ImmunologyFaculty of Veterinary MedicineSadat City UniversitySadatEgypt; ^2^ Department of Public HealthMicrobiology UnitCollege of Public Health and Health InformaticsQassim UniversityBuraidahSaudi Arabia; ^3^ Department of Veterinary MedicineCollege of Agriculture and Veterinary MedicineQassim UniversityBuraidahSaudi Arabia; ^4^ Department of Medical laboratoriesCollege of Applied Medical ScienceQassim UniversityBuraidahSaudi Arabia; ^5^ Department of Animal MedicineFaculty of Veterinary MedicineKafrelsheikh UniversityBuraidahEgypt; ^6^ Department of PhysiologyFaculty of MedicineZagazig UniversityZagazigEgypt

**Keywords:** Discrimination, Identification, MALDI Biotyper, MRSA, MSSA, *Staphylococcus* species

## Abstract

This study was designed to evaluate the ability of MALDI Biotyper (MBT) compared with Vitek^™^ 2 compact system for accurate identification of *Staphylococcus aureus* (*S. aureus*) and coagulase‐negative staphylococci (CNS) strains and discriminate methicillin‐sensitive *S. aureus* (MSSA) from methicillin‐resistant *S. aureus* (MRSA). Throughout Al‐Qassim region, Saudi Arabia, a total of 198 isolates of *S. aureus* (132 MSSA and 66 MRSA) and 44 CNS were collected from five dairy farms where the prevalence of staphylococcal mastitis was reported. The results produced by Vitek^™^ 2 compact system demonstrated that 123/132 MSSA isolates (93.18%), 61/66 MRSA (92.42%), and 37/44 CNS species (84.09%) were correctly identified. However; 130/132 MSSA (98.48%), 64/66 MRSA (96.96%), and 44/44 CNS (100%) were correctly identified by MBT with score ≥2. 00. The principal component analysis (PCA) dendrogram generated by MBT illustrated that the tested isolates were classified into two groups of *Staphylococcus* species at the distance level of 600. *S. aureus* isolates were found to be closely related with higher peak intensities in the mass of 3,993 Da, 4,121 Da and 5,845 Da were detected in MRSA, whereas, that were lost in MSSA. Conclusion: This study verified that MBT is an alternative powerful tool for precise identification and discrimination of *Staphylococcus* species.

## 1 Introduction

Bovine mastitis remains one of the most important complications among dairy farms all over the world from the diagnostic, economic, and public health related perspective (Benić et al., 2012; El Behiry et al., 2014; Preethirani et al., 2015). Several cases of mastitis in cattle are caused mainly by different types of bacteria, and the species of *Staphylococcus aureus* (*S. aureus*) and coagulase‐negative staphylococci (CNS) are considered the most common and important bacteria that cause mastitis, both subclinical and clinical form of mastitis among dairy farms (Cabral et al., 2004; Haveri, 2008; Vasudevan et al., 2003). Moreover, the presence of these pathogens in milk of animals raises a public health concern to the consumer due to its ability to yield several enzymes and enterotoxins which causes grave food poisoning (Johler et al., 2013).

In order to analyze outbreaks, the historically practiced methods of recognition and classification of *S. aureus* and CNS mostly by conventional ways based on the physiological and morphological characters are not accurate and laborious. In general, the morphological identification of bacteria is frequently shadowed by biochemical analysis of virulence genes as coagulase and thermonuclease (Wang et al., 2013). However, phenotypic and genotypic analyses are considered the most truthful methods for identification of *S. aureus* mastitis; these methods are laborious, expensive, and are not up till now been usually utilized in discriminating methicillin‐resistant *S*. *aureus* (MRSA) from methicillin‐sensitive *S. aureus* (MSSA). Although, MRSA was recognized **(**Koivula et al., 2007; Wolters et al., 2010 **)** and discriminated from MSSA due to spectral variations **(**Böhme et al., 2012; Du et al., 2002 **),** there is little evidence that has been presented regarding the use of matrix‐assisted laser desorption/ionization‐time‐of‐flight mass spectrometry (MALDI‐TOF‐MS) protein fingerprinting as a typing technique for grouping the different isolates of *S. aureus* at the strain level. Furthermore, one of the most common restrictions associated with the precise diagnosis of CNS is the absence of an exact, fast, and suitable technique that can discriminate the microbial species including this group (Cantekin et al., 2015; Koivula et al., 2007; Wang et al., 2013).

To facilitate the investigation of eruptions, the contagious isolates must be typed; on the other hand, nearly all molecular techniques are luxurious or labor‐intensive. For this reason, there is still a need for supplementary quick, low‐cost, and a precise technique for detection of various microorganisms of infectious diseases. In general, innovative techniques such as MALDI‐TOF‐MS for accurate and fast characterization of different microbes is considered an important step toward suitable prevention and control of communicable diseases in medical and veterinary diagnosis and currently are of major concern (El Behiry et al., 2014; Hays & Van Leeuwen, 2012; Sauer et al., 2008).

Formerly, the MALDI mass spectral imaging technique was developed as a tool for intact protein imaging of the tissue surface (Stoeckli et al., 2001; Stoeckli et al., 2002). Recently, a powerful mass spectral (MS) technique has developed the fields of proteomics, lipidomics, and metabolomics. This MS revolution has fortified researches to value the individual characterization and quantitation of biomolecules in living systems. In general, proteomics are defined as the identification and quantification of all the expressed proteins of a biological sample, with the aim of understanding their functions, their interactions, and their contribution to biological processes. Correspondingly, metabolomics has the goal to list and enumerate the entire range of metabolites. The developing field of lipidomics concentrates on the whole changes of lipid molecules (Ferreira et al., 2010).

Although the MS was recognized at the beginning of 1900s, its application was restricted to the chemistry sciences. Therefore, the progress of MALDI‐TOF‐MS in 1980s extended the scope of MS to different biological molecules such as proteins (Singhal et al., 2015). In an attempt to identify a cheaper and accurate method of identification, we employed protein fingerprinting using MALDI‐TOF‐MS as a diagnostic method for typing and fast detection of *Staphylococcal* species. MALDI‐TOF technology allows precise detection of numerous types of microorganisms in limited time, from 2 days to a few minutes, with a slight amount of bacteria needed for an accurate investigation (10^4^ to 10^6^ CFU). Mass spectral Identification of microbes by MALDI‐TOF‐MS can be used for a large variety of bacteria, yeasts, and molds (Liu et al., 2007; Pignone et al., 2006). Limited numbers of cells are enough for rapid and exact identification.

MALDI Biotyper (MBT) is a diagnostic method in which chemical substances are ionized into charged molecules and the mass charge ratio (m/z) is considered. Spectra ranging from 2,000 to 20,000 m/z were examined using the MBT system's automated control and the identified values of ≥2.000 specified species‐level identification, values ranging from 1.700 to 1.999 pointed to the identification of genus level, and scores of 1.700 showed no faithful identification (Chen et al., 2014; Cheng et al., 2015; Deng et al., 2014). Our current study aims to evaluate the performance of MBT compared with an automated colorimetric method using the Vitek^™^ 2 compact system in the identification of *S*. *aureus* and CNS species and discrimination of MSSA from MRSA strains isolated from milk of dairy cows suffering from mastitis to deliver a novel opportunity for fast, real identification, and control of serious staphylococcal mastitis.

## 2 Materials and Methods

### 2.1 Microorganisms

A total of 198 *S. aureus* isolates and 44 CNS were isolated from milk of mastitic cows during a survey carried out from November 2014 to December 2015. Isolation was carried out according to the recommendations described by the National Mastitis Council on investigation of milk samples. Preliminary identification and characterization were carried out by conservative approaches, for example, colony morphology (golden‐yellow pigmented colonies), positive for catalase and coagulase test and those that exhibited hemolytic characters were identified as *S. aureus* strains. Sensitivity of the isolates to methicillin was detected by agar disk diffusion test. *S. aureus* American Type Culture Collection (ATCC) 25923 (MSSA) and *S. aureus* ATCC 33591 (MRSA) were utilized as positive controls in each set of tests. All isolated cultures were stored at −80°C in a microbial cryopreservation system (MAST CRYOBANK^™^) till further investigations.

### 2.2 Isolation of genomic DNA

DNA was extracted from all *S. aureus* isolates using Nexttec^TM^ cleanColumn (nexttec GmbH Biotechnologie, Leverkusen, Germany). The extraction method was performed according to the manufacturer's guidelines to obtain a purified DNA.

### 2.3 PCR amplification

PCR amplifications were carried out with a triple of primers specific for the *nuc* gene, which encodes to specific regions of the thermonuclease of *S. aureus*, mecA gene, which is specific to methicillin resistance and 16*S rRNA,* a genus‐specific gene for staphylococcal species. The primers were shown in Table 1 and designed according to the sequences published in Biomers, Ulm, Germany.

**Table 1:** Oligonucleotide sequences and product length of *S. aureus* gene‐specific primers

Amplified gene	Forward PCR primer sequence (5′–3′)	Reverse PCR primer sequence (5′–3′)	Amplicon size (bp)
*Nuc*	TCAGCAAATGCATCACAAACAG	CGTAAATGCACTTGCTTCAGG	255
*mecA*	GGGATCATAGCGTCATTATTC	AACGATTGTGACACGATAGCC	527
*16S rRNA*	GTGCCAGCAGCCGCGGTAA	AGACCC GGGAACGTATTCAC	886

### 2.4 Colorimetric identification of *Staphylcoccus species* by Vitek^™^ 2 compact system

All preliminary identified isolates of *S. aureus* (MSSA and MRSA) and CNS were tested by Vitek^™^ 2 compact system (BioMe′rieux, France) according to the procedures provided by the company. The results were finally interpreted and tabulated automatically by the ID‐GPC library. The confidence value was expressed by seven various categories of results: excellent, very good, good, acceptable identification (only one result is delivered), low discrimination (if more than one result is recorded, the software recommends supplementary investigations to be achieved), or the data are inconclusive or unidentified.

### 2.5 Proteomic identification using MBT compass software

#### 2.5.1 Bacterial test standard (BTS) preparation

A quantity of 50 μl of standard solvent was added to the in vitro diagnostic product (IVD) BTS pellet and dissolved by pipetting up and down for 20 times at room temperature. The IVD BTS solution was then mixed for 5 min by pipetting up and down for 20 times and centrifuged at 13, 000 rpm for 2 min. 2 μl aliquots of the supernatant were pipetted into a separate screw cap, micro tubes and the rest was stored at −18°C.

### 2.6 Sample preparation

For preparation of the samples for MBT, a single fresh colony of overnight culture, incubated for 18–24 hr at 37°C was used for each isolate. Ethanol/formic acid extraction technique was performed as described by Bruker Daltonik, Germany. In brief, 1–2 colonies were transferred into a sterilized Eppendorf tube and mixed carefully in 300 μl of deionized water. A quantity of 900 μl of 100% ethanol was then added, the contents of the tube were judiciously mixed, and the tube was then centrifuged at 13, 000 rpm in a desktop centrifuge (centrifuge 5430, Eppendorf, USA) for 2 min. The liquid that lay above the sediment was discarded and the sediment was dried in air for at least 5 min. From 10 to 80 μl of formic acid (70%) was then supplemented according to the dried sediment size and mixed vigorously. An equivalent volume of acetonitrile, like formic acid, (70%) was then added and the mixture was centrifuged again at 13, 000 rpm for 2 min and then 1 μl of the liquid, floating on the surface above the precipitate was placed onto 96‐well target plate and permitted to dry at 25°C. Consequently, each sample was inoculated with 1 μl of matrix solution, which formed a saturated solution of α‐cyano‐4 hydroxy‐cinnamic acid (HCCA) in 50% acetonitrile and 2.5% trifluoroacetic acid (final concentration: 10 mg HCCA/ml) purchased from Sigma Aldrich (# 19182) and dried in air at 25°C. The target plate was successively presented into the MBT device for direct measurement and data interpretation via FlexControl software with Compass Flex Series version 1.3 software and a 60‐Hz nitrogen laser (337 nm wavelengths). All tested samples were blinded and run in duplicates for obtaining accurate identification.

### 2.7 Data analysis

In the range from 0 to 3, the score value of unknown spectrum was detected by matching the unknown spectrum with a recognized spectrum from the MBT taxonomy. The precision of the species identification was determined as described by the manufacturer procedures of Bruker Daltonics, Germany. The extremely accurate identification was carried out by MBT, when the log score was in the range from 2.30 to 3.00; however, species and probable genus identification were detected in the range from 2.000 to 2.299 and 1.700 to 1.999, respectively. In addition, the log score range from 0.000 to 1.699 meant that the identification was not reliable. The different spectra produced by compass software were evaluated in a m/z range between 2, 000 Da and 20, 000 Da. The acceptance criteria were based on 50 laser shots per spot. To discriminate between individual strains, an equivalent number of spectra per strain was required: 25 spectra were selected and mathematical testing of the datasets was done on the basis of Principal Component Analysis (PCA) and the results were demonstrated in a three‐dimensional (3d) score plot, automatically created by the software. According to the MBT database, containing 132 main spectra of staphylococcus species and subspecies, the PCA dendrogram setting was utilized to group species. Moreover, a dendrogram was created from the minimal spanning tree (MSP) dataset. The MSP dendrogram was created according to the assessment of the principal spectra of analyzing the strains. Firstly, each main spectrum of the MBT library was compared with each of the other main spectra resulting in a matrix of cross‐wise identification scores. This matrix was utilized to estimate the distance values for each pair of main spectra. According to these values, a dendrogram was produced by means of MBT software.

## 3 Results

### 3.1 Identification of *S. aureus* and CNS strains/colorimetric identification

198 *S. aureus* and 44 CNS isolates were evaluated by the Vitek^TM^ 2 compact system for colorimetric identification. According to the interpreted results in Table 1, a total 184 out of 198 *S*. *aureus* strains were correctly identified as 123/132 of MSSA isolates (93.18%) and 61/66 of MRSA (92.42%), while 37/44 of CNS strains were correctly identified as 10/13 (76.92%) of *Staphylococcus hominis* (*S. hominis*), 10/12 (83.33%) of *Staphylococcus chromogenes* (*S. chromogenes*), 7/9 (77.77%) of *Staphylococcus simulans* (*S. simulans*), 6/6 (100%) of *Staphylococcus warneri* (*S. warneri*), and 4/4 (100%) of *Staphylococcus sciuri* (*S. sciuri*).

### 3.2 Species identification of *S. aureus* and CNS isolates by MBT

In this study, the tested isolates were analyzed by MBT and the resulting spectra were compared with the spectra in the library delivered with the MBT compass software. A distinctive analysis of numerous isolates of *S. aureus* and CNS isolated from the milk of apparently healthy cows and cows that showed symptoms of mastitis were performed by MBT. From 10 to 20 prominent ion peaks were noticed in the line spectra from an area ranging from 2, 000 to 16, 000 Da, with a higher strength peaks observed between 3, 000 and 10, 000 Da that matched with six MSSA reference strains in the Bruker taxonomy (*S. aureus* ssp aureus ATCC 25923, *S. aureus* ssp aureus ATCC 29213, *S. aureus* ssp aureus ATCC 33862, *S. aureus* spp aureus DSM 20231, *S. aureus* spp aureus DSM 799, and *S. aureus* spp aureus DSM 346), four MRSA reference strains (*S. aureus* ATCC 33591 THL, *S. aureus* ssp aureus DSM 4910, *S. aureus* spp aureus DSM 20232, and *S. aureus* spp aureus DSM 3463), and six reference strains of CNS (*S. hominis* Mb 18788 _1 CHB, *S. hominis* 18 ESL, *S. simulans* DSM 20323, *S. chromogenes* DSM 20454T, *S. sciuri* ssp carnaticus DSM 15613T, and *S. warneri* DSM 30728).

In this study, 130/132 (98.48%) of MSSA and 64/66 (96.96%) of MRSA isolates were correctly identified with a score value ranging from 2.300 to 3.000 for 72 isolates of MSSA and 48 isolates of MRSA, 57 isolates of MSSA and 16 isolates of MRSA were also properly recognized with a score value from 2.000 to 2.299. Whereas; five isolates of *S. aureus* (3 MSSA and 2 MRSA) were identified at the genus level with log score ranging from 1.7 to 1.99 (Table 2). While; 29 isolates of the CNS were correctly identified with score value ≥2.30 and 15 isolates were correctly identified with the score ≥2.00. The *S. aureus* and CNS isolates were identified by matching their spectral profiles with MBT library, which contains over 300 strains of 60 genera from the national collection of type cultures, including 18 species of the genus *Staphylococcus*.

**Table 2:** Performance of the Vitek^™^ 2 compact system and MALDI Biotyper (MBT) for identification and discrimination of *S*. *aureus* and CNS isolated from bovine mastitis

Isolate	Total	Vitek^™^ 2 compact		MBT	
		Number	CI (%)	Number	CI (%)
*S. aureus*					
MSSA	132	123	93.18	130	98.48
MRSA	66	61	92.42	64	96.96
CNS					
*S. hominis*	13	10	76.92	13	100
*S. chromogenes*	12	10	83.33	12	100
*S. simulans*	9	7	77.77	9	100
*S. warneri*	6	6	100	6	100
*S. sciuri*	4	4	100	4	100

CI, correctly identified rate.

**Table 3:** Identification of 198 strains of *S. aureus* and 44 of CNS by MBT

Category	Score range	No. of *S. aureus*		No. of CNS
		MSSA	MRSA	
1	2.3–3	72	48	29
2	2–2.29	57	16	15
3	1.7–1.9	3	2	0
4	0–1.6	0	0	0

**Figure 1:**
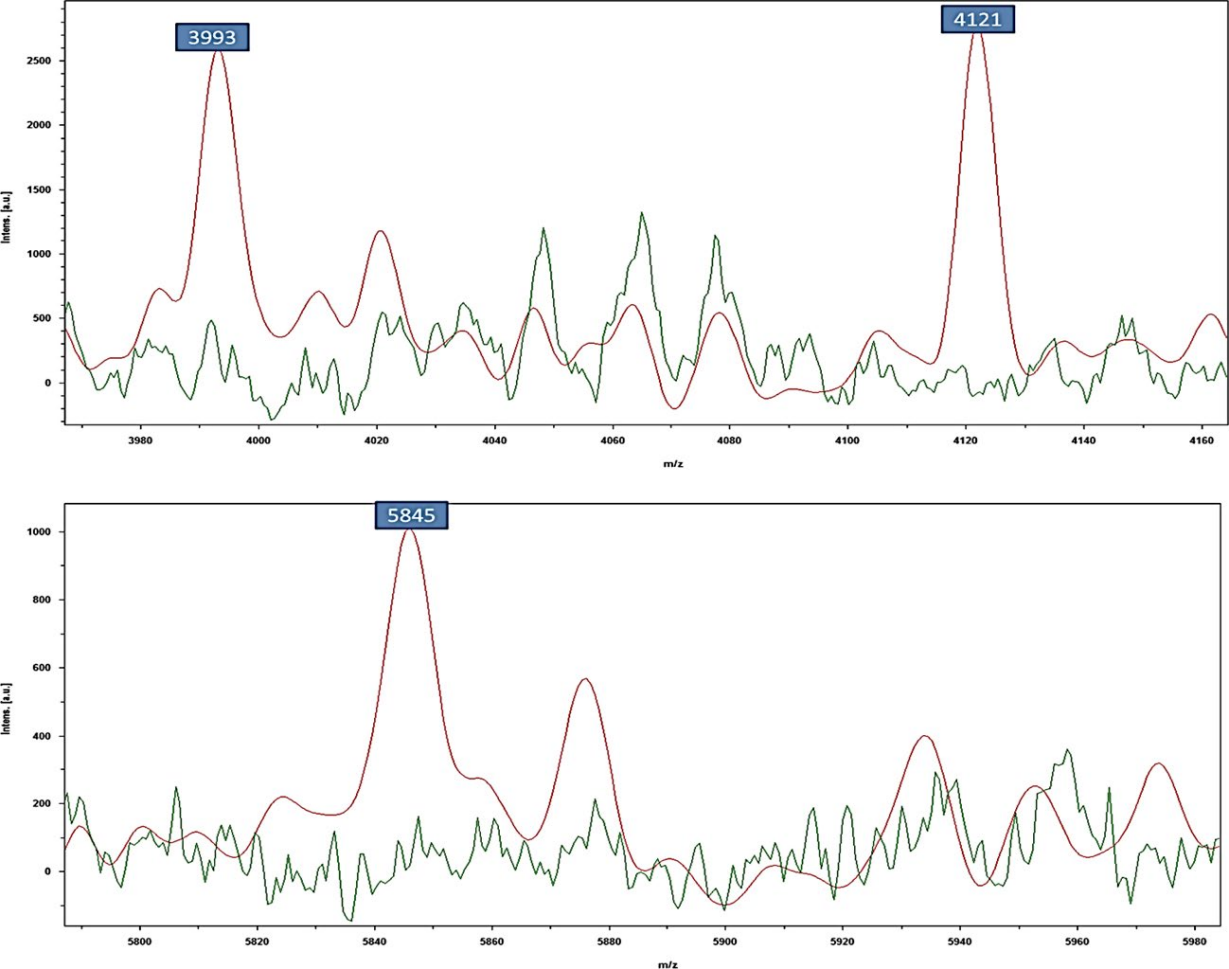
Higher peaks intensity (3,993 Da, 4,121 Da, and 5,845 Da) were detected in MRSA (red), whereas, they were missed in MSSA (green)

**Figure 2:**
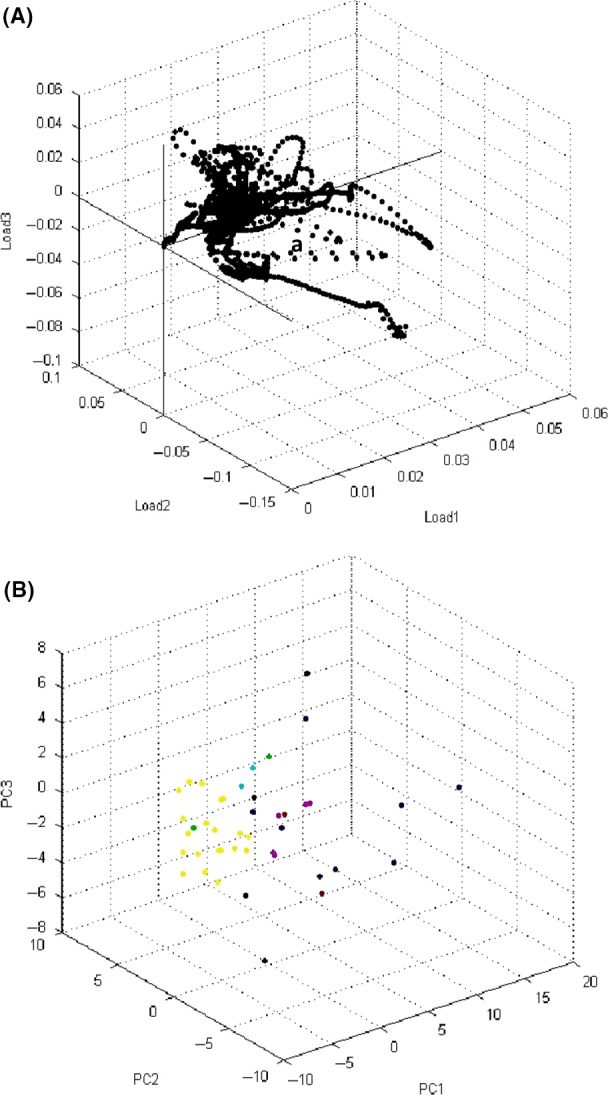
The dimensional image from PCA shows several spectra for *S. aureus* and CNS milk isolates (A) The dots display the matching spatial scattering state of the variance peaks in loading mode. Each dot indicates the intensity value of the peaks. The peaks were different up to the loading value corresponding to the loading (Loading1, Loading 2, Loading 3) model in three binary images (B) The classification of strains in the first three principal component model (PC1, PC2, PC3)

**Figure 3:**
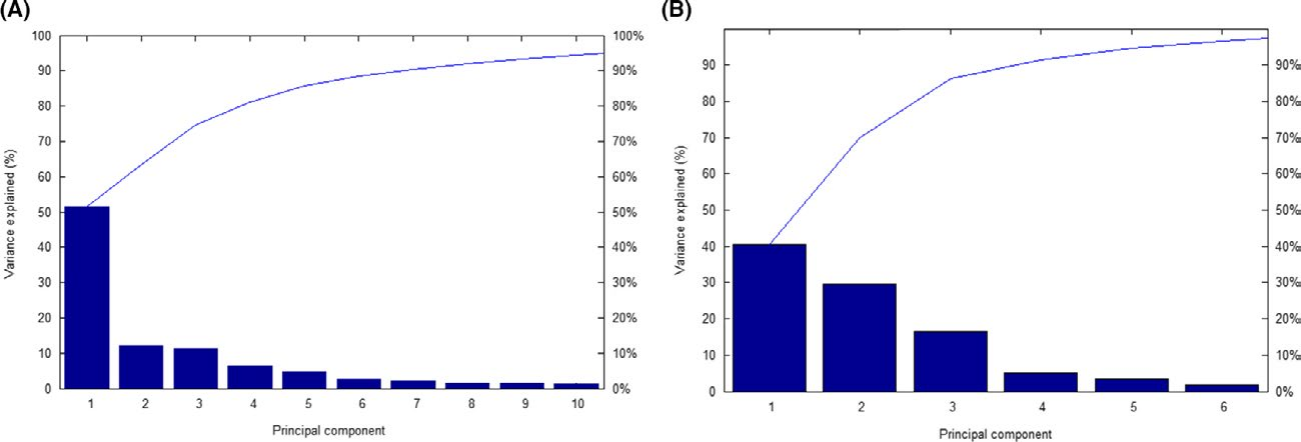
The influence of principal components to the profiling classification in plot of proportion explained variance of principle component (A) The contributions of PC1, PC2, and PC3 of 10 principle components were approximately 52%, 13%, and 10% for *S. aureus* strains (B) The contributions of PC1, PC2, and PC3 for five principal components were nearly 40%, 29% and 18% for CNS

Depending upon visual examination of mass regions, numerous variations were detected to differentiate between MSSA and MRSA. It was noticed that the clearest area of manifold signals, in the range from 3, 800 to 5, 900 Da, showed various intensities between MSSA and MRSA strains. Consequently, the peaks considered as an important signal to discriminate between MRSA and MSSA were reliably observed by the visualizing features. The particular views were magnified into the region of about 3,993 Da, 4,121 Da, and 5,845 Da, correspondingly. In these cases, the peaks of interest displayed various signal intensities between MRSA and MSSA. A higher peak intensities in the mass of 3,993 Da, 4,121 Da, and 5,845 Da (Fig. 1) was noticed in MRSA than in MSSA. The principal component analysis (PCA) is considered an additional mathematical tool used in the analyzed datasets to visualize the degree of similarity and diversity of the protein spectra. Moreover, the PCA diminishes the differences in a complex dataset according to the various statistical tests. Several protein spectra of *S. aureus* and CNS strains were demonstrated in three‐dimensional (3d) PCA in (Fig. 2A). Each spot referred to a protein spectrum and the different colors illustrate the considered cluster involvement in which each spot is considered as one measured protein spectrum profile. The resulting of the 3d PCA cluster view of the peaks of all strains of *S. aureus* showed that most of the peaks were closely related and matched together (Fig. 2B). Concerning the calculation of the PCA algorithm, every peak could get loading values which originate from the calculation of PCs. In this study, each peak was specified with three loading values originated from three PCs (PC1, PC2, and PC3) calculation. Briefly, the impacts of PC1, PC2, and PC3 to the generation of the profile in a percentage conspiracy of the difference elucidated, were about 52%, 13%, and 10% for *S*. *aureus* and 40%, 29%, and 18%, correspondingly for CNS (Fig. 3).

**Figure 4:**
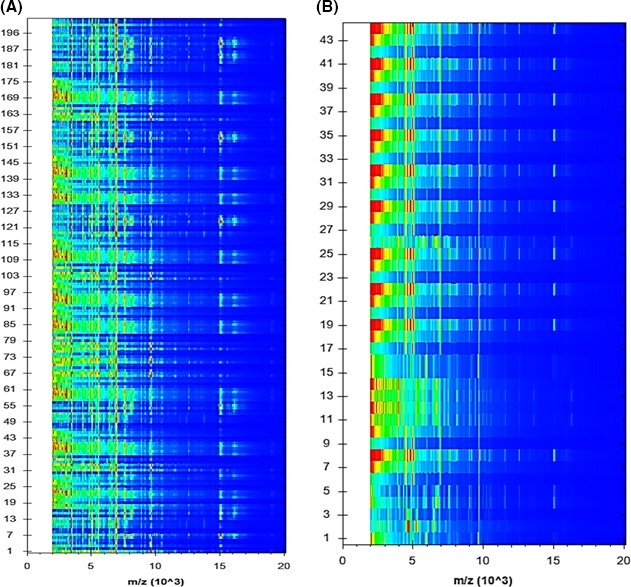
Gel view of protein spectra for 198 *S. aureus* and 44 CNS isolates. The varied color of spots was the assembly of protein spectra with various contents

**Figure 5:**
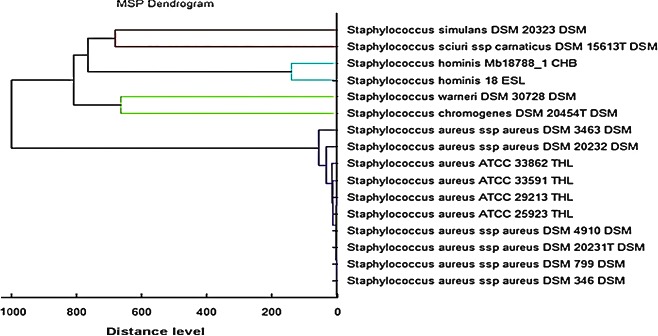
The MSP dendrogram for 198 *S. aureus* and 44 CNS in which the strains of *S. aureus* showed a close relation with 10 reference strains in the Bruker taxonomy, especially with *S. aureus* ATCC 33862, *S. aureus* ATCC 33591, *S. aureus* ATCC 29213, *S. aureus* ATCC 25923, *S. aureus* DSM 4910, *S. aureus* DSM 20231, *S. aureus* DSM 799, and *S. aureus* DSM 346. Additionally, another close relation was found between *S. hominis* Mb 18788_1 CHB and *S. hominis* 18 ESL

An effective gel view over the viewing representation of data derived from 198 well‐characterized strains of *S. aureus* and 44 CNS is revealed in Figure 4. All single spectra are demonstrated on a density scale and as can be seen from this figure, the intensity distribution of the appropriate signals in the diverse specimens. To elucidate if the MBT software could discriminate clonally related strains at the species level, spectra from 198 well‐identified *S. aureus* strains were analyzed as described above. The spectra were then utilized to create a new cross‐wise minimal spanning tree (MSP) dataset (Fig. 5). It was observed that the results of dendrogram showed that the analyzed isolates of *S. aureus* were closely related to 10 reference strains of *S. aureus* and different from CNS. The MSP dendrogram showed the adjacent relation between the field isolates and some reference strains of *S. aureus,* especially *S. aureus* ATCC 33862, *S. aureus* ATCC 33591, *S. aureus* ATCC 29213, *S. aureus* ATCC 25923, *S. aureus* DSM 4910, *S. aureus* DSM 20231, *S. aureus* DSM 799, and *S. aureus* DSM 346. Another close relation between *S. hominis* 18 ESL and *S. hominis* Mb 18788_1 CHB was found. It was confirmed that identification and discrimination of *S. aureus* and CNS strains by MBT are more precise than colorimetric method, with an accuracy of 98.57% and 100%, respectively. As regards the time factor, mass spectral identification method takes less time to be carried out, approximately 90 min to investigate a full 96‐spot target plate. Consequently, MALDI‐TOF‐MS was considered as a rapid, economic, and extremely precise technique for identification and discrimination of various microorganisms. The PCR technique was used to confirm all strains of *S. aureus* with low score value ((1.700–1.999) that presented by MBT. The results of PCR indicated that all strains of MSSA and MRSA were positive to nuc and mecA genes and identified as *S. aureus*.

## 4 Discussion

Most of the pathogens present in the environment are difficult to control as they acquire latest means of antibiotic resistance from other organisms through horizontal transfer (Barlow, 2009). Identification of the source organism in time and proper removal is the only key to control the spread of bovine infections. A victorious management strategy for different forms of bovine mastitis can be recognized with a successful controlling scheme for various dairy farms and precise detection of germs causing mastitis (Deb et al., 2013; El Behiry et al., 2012). *S. aureus* and CNS have become public bacterial pathogens of bovine mastitis, predominantly of those being resistant to methicillin. Failing to properly detect *Staphylococcus* species in the initial phases of intramammary infections may result in a more prolonged and expensive treatment and recovery. Traditional approaches for identifying staphylococcal mastitis are sluggish, expensive, and are hardly reliable **(**Johler et al., 2013; Liu et al., 2007). Therefore, in this context, precise, fast, and early identification of *S. aureus* (MSSA and MRSA) and CNS strains isolated from milk of mastitic cows are tremendously significant.

A simple and accurate colorimetric method using the Vitek^TM^ 2 compact system for identifying the various species of bovine staphylococcal isolates would have significance for identification of species which associated with the variable forms of clinical severity of mastitis and with raised somatic cell counts. Such technique would permit the researchers to faster identification of *Staphylococcus* species which responsible for mastitis instead of having to submit specimens to an animal diagnostic laboratory for investigation. In this study, 198 *S. aureus* (132 MSSA and 66 MRSA) isolates and 44 CNS isolates from apparently healthy cows and cows that showed signs of mastitis were preliminarily identified and differentiated by tube coagulase test and then tested by the Vitek^™^ 2 compact system. According to the obtained results, 37/44 isolates of CNS (84.09%) and 184/198 isolates of *S. aureus* (92.92%) were correctly identified. Similar findings agreed with that recorded by previous study (Spanu et al., 2003), which assessed the capability of the VITEK 2 system for fast detection of 130 *S. aureus* and 275 CNS isolated from blood cultures and illustrated that 90.5% of CNS and 99.2% of *S. aureus* strains were correctly identified. Moreover, another study recorded the performance of Vitek 2 System on 11 *S. aureus* isolates and stated that the percentage of identification was 100% on genus and species levels (Da Silva Paim et al., 2014).

In our study, the Vitek^™^ 2 compact system established equivalent rates of correctness in the detection of mastitis caused by staphylococcus species. Therefore, microorganisms with low detection levels included a very restricted number of strains to provide a valuable indication for improvement by Vitek^™^ 2 compact system. The fact that staphylococcal isolates which were not identified properly could be described by their sluggish metabolism, leading to indistinct results in the reaction wells. Similar findings agreed with that recorded previously (Da Silva Paim et al., 2014; Ligozzi et al., 2002; Pfaller & Herwaldt, 1988), who evaluated the other automated identification systems used for identification of various staphylococcus species. Although, several gram‐positive microorganisms were correctly identified by Vitek 2 Compact system, some restrictions of this technique lead to failure of detection of uncommon bacteria. Subsequently, supplementary tools may be compulsory to precise detection of certain strains at the species level (Carbonnelle et al., 2007).

MALDI‐TOF‐MS therefore, represents one of the major, fast and correct tool for identification and typing of various microorganisms isolated from clinical and subclinical samples (Carbonnelle et al., 2007; El Behiry et al., 2014; Singhal et al., 2015). In our study, detection and typing of *S. aureus* and CNS strains were carried out by proteomic analysis and the frequency of precise identification at the genus and species levels was 194/198 (97.97%) and 44/44 (100%), respectively, with the score level ranging from 2,000 to 3,000 Da. Parallel results were recorded previously (Loonen et al., 2012), who identified CNS isolates isolated from clinical cultures by five techniques: MALDI‐TOF‐MS, Vitek2 System, Staph ID 32 API system, 16S rRNA gene sequencing, and *tuf* gene sequencing. They found that the highest identification rate (99.3%) was detected by MALDI‐TOF‐MS.

In this context, the lower preliminary values for five *S. aureus* strains at the species level with the score ranging from 1.700 to 1.999 could be associated with the preparation of samples and the quantity of matrix in the sample in the first measurement were not ideal. Therefore, it is very important to use other conventional techniques like a nuc‐based PCR test as a supplemental method to approve the suspected results obtained by MBT (Clinical and Laboratory Standards Institute (CLSI), 2013). The data of the current research were analyzed by MBT and demonstrated that the most spectral peaks for the tested *S. aureus* strains were ranged from 3,000 to10,000 Da. This range is similar to several studies on microbial detection using MBT (Barreiro et al., 2010; Lartigue et al., 2009; Silva et al., 2015). In contrast, this range was somewhat wide compared with the former studies, which noticed that most of the spectral peaks ranged from 800 to 3,500 Da (Edwards‐Jones et al., 2000; Walker et al., 2002). These variations in the mass, charge range of spectral peaks could be explained by the variance in preparation of the sampling methods. Numerous investigators have examined MBT typing profiles. Certain studies confirmed the association between MBT profiles created by both MRSA and MSSA. In this study, the selected peaks at the masses of 3,993 Da, 4,121 Da, and 5,845 Da gave strong confirmation for the discrimination between MRSA and MSSA. The period to obtain the results created by MBT was obviously less than that produced by conservative and molecular approaches.

Despite the capital cost and maintenance of MALDI‐TOF–MS, it is considered as one of the potential drawbacks (Chen et al., 2013; Neville et al., 2011; Pavlovic et al., 2013). This technique represents a powerful new tool that has the potential to replace conventional identification tools for a majority of routine isolates encountered in microbiology laboratories. The most prominent variations between the MALDI‐TOF‐MS and traditional identification techniques are detected in the expected time and cost needed for sample identification. The cost of bacterial identification by MALDI‐TOF‐MS was estimated to represent approximately €1.43/sample while the costs of conventional identification methods, about €4.6–8.23/sample (Bizzini et al., 2010; Seng et al., 2009). Furthermore, another study indicated that the reagents compulsory for phenotypic identification using modern automated instruments cost about $10 per isolate, whereas, the required reagents for MALDI‐TOF MS do not exceed $0.50 per sample (Cherkaoui et al., 2010).

Furthermore, the proteomic identification was fast and took about 30 min per isolate from target plate to obtain the final results, and nearly 2 hr to investigate a full 96‐spot target plate. Therefore, when we compared the MALDI‐TOF‐MS with the colorimetric method in this study, it was noticed that the utilization of the MALDI‐TOF‐MS for a routine identification of various *Staphylococcus* strains is reliable which is characterized mainly by simple procedures, large amount of sampling, high accuracy, more sensitivity, and reproducibility.

Through this study, we verified that identification of *S. aureus* and CNS strains isolated from mastitic cows by MBT combined with compass software was faster, economic, and more precise than The Vitek 2 compact system and also was successfully discriminated MSSA from MRSA according to the peak intensities. Consequently, the opportunity of solving the problem of clinical and subclinical bovine mastitis was possible by precise and early diagnostic method.

## Funding Information

This study was supported by grant number 13‐BIO1683‐09 from the National Science, Technology and Innovation Plan (NSTIP), Saudi Arabia.

## Conflict of interest

The authors declare that there is no conflict of interests regarding the publication of this paper.
